# Investigation of psychiatric symptoms, dissociative experiences, and childhood trauma in allergic rhinitis, and evaluation of the clinical relationship between these factors

**DOI:** 10.3389/fpsyt.2026.1900556

**Published:** 2026-09-16

**Authors:** Mustafa Altintas, Süleyman Korkut, Deniz Şarlak, Erdinc Ozturk, Osman Celbiş

**Affiliations:** 1 Department of Otorhinolaryngology, Antalya Training and Research Hospital, University of Health Sciences, Antalya, Türkiye; 2 Department of Psychiatry, Antalya Training and Research Hospital, University of Health Sciences, Antalya, Türkiye; 3 Department of Psychology, Mugla Sitki Kocman University, Muğla, Türkiye; 4 Institute of Forensic Sciences and Legal Medicine, Depatment of Social Sciences, Istanbul University – Cerrahpaşa, Istanbul, Türkiye; 5 Alanya Alaaddin Keykubat Universitesi, Antalya, Türkiye

**Keywords:** allergic rhinitis, anxiety, childhood trauma, depression, dissociation

## Abstract

**Background:**

In this study, we aimed to evaluate levels of depression and anxiety symptoms, childhood trauma, and dissociative experiences in patients with allergic rhinitis (AR) and compare them with healthy controls. Additionally, investigate the association between childhood trauma, depressive symptoms, and dissociative experiences in AR patients.

**Methods:**

The study sample consisted of 152 adults (76 AR patients and 76 healthy controls) who presented to the otorhinolaryngology outpatient clinic. Data were collected using a personal information form and standardized self-report scales for childhood trauma (Childhood Trauma Questionnaire – CTQ-28), anxiety and depression (Hospital Anxiety and Depression Scale – HADS), and dissociative experiences (Dissociative Experiences Scale – DES).

**Results:**

Significant differences were found between the patient and control groups in terms of all parameters (p<0.05). The patient group showed significantly higher levels of childhood trauma, anxiety and depressive symptoms, and dissociative experiences. Correlation analyses revealed a significant correlation between childhood trauma and both depressive symptoms and dissociative experiences in AR patients. Furthermore, regression analysis showed that childhood trauma significantly contributed to the severity of depression in AR patients, while the presence of dissociative symptoms further increased the risk of depression.

**Conclusions:**

This study presents evidence of a positive association between childhood trauma, depression, and dissociative experiences in allergic rhinitis, highlighting their potential roles as risk factors for the condition. Therefore, psychiatric evaluation is crucial and necessary for treatment success in AR patients.

## Introduction

Allergic rhinitis (AR) is a prevalent chronic inflammatory disease of the nasal and paranasal sinus mucosa, affecting a substantial proportion of the population. Its prevalence is higher in industrialized societies, ranging between 10% and 40% (1, 2). AR has become a significant public health concern due to its growing incidence and associated economic burden (3). The main symptoms include sneezing, nasal discharge, congestion, nasal itching, headache, and red, watery, itchy eyes. These symptoms can lead to impaired concentration during the day, chronic fatigue, sleep disturbances, reduced productivity, unemployment, and negative emotional states such as depression and anxiety (4, 5) Owing to its chronic course and recurrent airway obstruction, AR may negatively impact quality of life and increase the risk of mental health disturbances. Indeed, the prevalence of anxiety and depression is higher among patients with AR compared to healthy individuals (6, 7).

Many studies have indicated that psychiatric problems play a significant role in allergic diseases, and that psychological stress factors can trigger allergic diseases (8–10). In relation to our study, it has been reported that AR patients exhibit increased anxiety, obsessive traits, and neurotic tendencies compared to unaffected individuals (11). Such psychological factors may be fundamentally rooted in early-life traumatic experiences and dissociative tendencies. There is considerable evidence that childhood psychological trauma is an important factor in the development of dissociation (12, 13). Traumatic experiences in childhood can have lasting effects on psychological functioning into adulthood. Dissociative symptoms may emerge following traumatic events such as abuse, accidents, or natural disasters, and trauma-related dissociation can lead to fragmentation of mental functions often observed in post-traumatic stress disorder. This dissociative “splitting” serves as a defense mechanism, it protects an individual from the immediate full impact of trauma by partitioning off overwhelming memories and emotions, postponing their processing (14).

To the best of our knowledge, this study is the first to comprehensively evaluate childhood trauma and dissociative experiences and examine the association between them in the AR. By filling this gap in the literature, our aim is to present new findings in our study that can guide future research.

In summary, the aim of this study is to evaluate the levels of depression and anxiety symptoms, childhood trauma, and dissociative experiences in the AR patients and compare them with healthy controls. Additionally, investigate the association between childhood trauma, depressive symptoms, and dissociative experiences in the AR.

## Methods

### Participants and procedures

The sample consisted of 152 volunteers over the age of 18 (76 patients with AR and 76 healthy controls). Patients were selected from individuals presenting to the Otorhinolaryngology outpatient clinic. A non-probability convenience sampling method, “easy sampling”, was used to enroll cases. The Allergic Rhinitis and its Impact on Astma (ARIA) guidelines were followed throughout the study. Patients were included if they presented with at least two of the following symptoms at examination: sneezing episodes, rhinorrhea (runny nose), nasal congestion, or itching of the eyes, nose, or palate. The diagnosis of AR was corroborated through the positive allergy skin tests. The Control group consisted of healthy participants (hospital staff) with no clinical signs of AR; all healthy controls (HCs) had negative results on allergy skin testing to rule out asymptomatic allergic sensitization, no psychiatric disorder, from the similar age and gender with the patient group.

Patients with perennial (year-round) AR were required to have symptoms for at least six months, and those with seasonal AR should have had symptoms during at least two allergy seasons. A nasal endoscopy was performed to evaluate intranasal findings (e.g., turbinate, color and edema, nasal secretions) further confirming the diagnosis of AR in patients.

Individuals were excluded if they had used any antihistamines, anti-inflammatory drugs, topical or systemic steroids, immunosuppressants, antidepressants, or antihypertensive medications in the last 3 months (to avoid confounding effects of treatment), had upper or lower respiratory infections in the last month), have chronic systemic diseases that could lead to rhinitis.

An experienced psychiatrist evaluated the psychological status of participants. Informed consent was obtained from the participants. The study was conducted in accordance with the principles of the Helsinki Declaration and the approval of the Local Ethical Committee was obtained (The Health Sciences Clinical Research Ethics Committee of Inonu University – Protocol no: 2018/22-10).

### Assessment tools

#### Personal information form

A semi-structured personal information form (PIF), developed by the authors, was used to collect sociodemographic data.

#### Hospital Anxiety and Depression Scale (HADS)

The Hospital Anxiety and Depression Scale (HADS) is a widely used self-report instrument designed to screen for anxiety and depression symptoms in patients in hospital or outpatient settings. The validity and reliability of the Turkish version of the Hospital Anxiety and Depression Scale, originally developed by Zigmond (15), were established by Aydemir et al. (16). The scale consists of 14 items, with 7 items assessing anxiety (HAD-A subscale) and 7 assessing depression (HAD-D subscale). In the original Turkish validation, Cronbach’s alpha reliability coefficients were reported as 0.85 for the anxiety subscale and 0.78 for the depression subscale. In the present sample, the internal consistency (Cronbach’s alpha) was 0.79 for the anxiety subscale and 0.77 for the depression subscale.

#### Dissociative Experiences Scale (DES)

The Dissociative Experiences Scale is a self-report instrument developed to quantify the frequency of dissociative experiences (DES) (17). Participants rates each of the 28 items from 0 to 100, indicating the percentage of time they experience each dissociative symptom. A comprehensive Turkish adaptation and validation of the DES was conducted by Yargıç et al. (18) The Turkish version of the DES has demonstrated high validity and reliability comparable to the original, with a reported Cronbach’s alpha of 0.91 and test–retest correlation of r=0.78 in its validation study. In our study, the DES showed an internal consistency of α=0.78.

#### Childhood Trauma Questionnaire (CTQ)

Childhood trauma was assessed using the Childhood Trauma Questionnaire (CTQ) (19), a widely used retrospective self-report scale that inquires traumatic experiences during childhood and adolescence. The Turkish version of this scale was adapted and validated by Şar et al. (20). This scale consists of 28 items in total and each item is rated from 1 (never) to 5 (very often). The scale provides five distinct subscale scores covering childhood sexual, physical, and emotional abuse and, emotional and physical neglect, together with a total score. In the adaptation study, the internal consistency coefficient of the scale was 0.93, and the test – retest reliability was 0.90. The coefficient of internal consistency of the scale in this study was determined to be 0.87.

#### Statistical analysis

Data from the PIF, CTQ, DES, and HADS were analyzed using IBM SPSS Statistics for Windows, Version 22.0. Descriptive statistics (frequencies, means, standard deviations) were calculated to summarize sample characteristics. Group differences between the AR and HCs were examined using independent-samples *t*-tests for continuous variables. The Chi-square test (χ2) was used to analyze the relationship between categorical variables. Pearson correlation analyses were performed to evaluate the relationships between childhood trauma scores and psychological measures (depression, anxiety, dissociation). A hierarchical multiple regression analysis was conducted (in the patient group) to identify significant predictors of depression scores. A 95% confidence level and a significance threshold of α=0.05 were used for all hypothesis tests.

## Results

### Sample characteristics

**Table 1** displays the distribution of AR cases and HCs by sociodemographic variables. In the AR group (n = 76), 69.74% of participants were female and 30.26% were male; 32.89% were aged between 20 and 30 years, 56.58% were married, 44.74% had a high school level of education, 48.68% were employed, 72.37% hadn’t consulted a psychiatrist, and 63.16% didn’t report any chronical disease. In the control group (n = 76), 65.79% were female and 34.21% were male; 31.58% were aged between 20–30 years, 71.05% were married, 36.84% had completed high school, 46.05% were employed, 68.42% hadn’t taken psychiatric treatment, and 61.84% didn’t report any chronical disease. There were no statistically significant difference between groups in terms of the distribution of age (p=0.379) and gender (p=0.729) (p>0.05).

**Table 1:** Sociodemographic characteristics of the groups.

	Allergic Rhinitis	Control Group
Gender		
Female, n (%)	53 (69.74)	50 (65.79)
Male, n (%)	23 (30.26)	26 (34.21)
Age		
20–30 years old, n (%)	25 (32.89)	24 (31.58)
31–40 years old, n (%)	23 (30.26)	17 (22.37)
41–50 years old, n (%)	17 (22.37)	16 (21.05)
Over 51 years old, n (%)	11 (14.47)	19 (25.00)
Marital status		
Married, n (%)	43 (56.58)	54 (71.05)
Single, n (%)	33 (43.42)	22 (28.95)
Education status		
Primary, n (%)	12 (15.79)	18 (23.68)
Middle school, n (%)	30 (39.47)	30 (39.47)
High School college, n (%)	34 (44.74)	28 (36.84)
Working condition		
Employed, n (%)	37 (48.68)	35 (46.05)
Unemployed, n (%)	39 (51.32)	41 (53.95)
Psychiatric History		
No, n (%)	55 (72.37)	52 (68.42)
Yes, n (%)	21 (27.63)	24 (31.58)
Chronic Disease		
No, n (%)	48 (63.16)	47 (61.84)
Yes, n (%)	28 (36.84)	29 (38.16)

### Group differences in trauma and psychological measures

The AR group demonstrated a total mean score of 68.90 ± 5.50, with subdimension scores of 11.27 ± 2.21 for emotional abuse, 12.68 ± 2.56 for physical abuse, 10.90 ± 2.07 for sexual abuse, 20.77 ± 1.98 for emotional neglect, and 13.26 ± 1.20 for physical neglect. Among the cases, HADS-A and HADS-D scores averaged 11.03 ± 3.22 and 10.43 ± 3.36, respectively, while the DES total score had a mean value of 15.98 ± 4.69. In the control group, the overall mean score was 26.25 ± 3.24, while subscale analyses revealed mean scores of 12.76 ± 1.84 for emotional abuse, 3.08 ± 1.03 for physical abuse, 5.28 ± 1.27 for sexual abuse, 12.76 ± 1.84 for emotional neglect, and 6.07 ± 1.07 for physical neglect. Accordingly, a statistically significant difference was found between the childhood trauma scores of patients with AR and HCs (t= 1.85; p<.05). The mean score of HADS-A in the HCs was 9.45 ± 1.01 and 8.04 ± 1.12, respectively, and the mean DES total score was 7.23 ± 3.24. Accordingly, a statistically significant difference was found between the mean HADS-D and HADS-A scores of patients with AR and those of the HCs participants (t=.72; p<.05) (**Table 2**).

**Table 2:** Comparing the mean scores of the scales among the groups.

Scales	Case Group X̄ SD	Control Group X̄ SD	t	p
Childhood trauma subdimensions				
Emotional abuse	11.27 (2.21)	8.71 (1.61)	1.081	0.02
Physical abuse	12.68 (2.56)	3.08 (1.03)	1.78	0.03
Sexual abuse	10.90 (2.07)	5.28 (1.27)	.71	0.04
Emotional neglect	20.77 (1.99)	12.76 (1.84)	.52	0.04
Physical neglect	13.26 (1.20)	6.07 (1.07)	1.28	0.05
Total childhood trauma	68.90 (5.50)	26.25 (3.24)	1.85	0.02
Dissociative experience scale	15.98 (4.69)	7.23 (3.42)	.31	0.03
Hospital anxiety and depression scale-depression	10.43 (3.36)	8.04 (1.12)	.72	0.02
Hospital anxiety and depression scale-anxiety	11.03 (3.22)	9.45 (1.01)	1.28	0.04

### Correlation and regression analysis

**Table 3** summarizes the relationship between childhood trauma in the cases and both depressive symptoms and dissociative experiences. A positive and statistically significant correlation was observed between HADS-D scores and all dimensions of the CTQ, as following, emotional abuse (r =0.342, p<0.05), physical abuse (r =0.241, p < 0.05), sexual abuse (r =0.310, p<0.05), emotional neglect (r=0.252, p<0.05), and physical neglect (r=0.281, p<0.05). Also, the total CTQ score showed a positive and statistically significant relationship with HADS-D score (r=0.442, p<0.05). Positive and statistically significant relationships were determined between DES scores and the emotional abuse (r=0.381, p<0.05), physical abuse (r=0.158, p<0.05), sexual abuse (r=0.256, p<0.05), emotional neglect (r=0.397, p<0.05), and physical neglect (r=0.389, p<0.05), as well as with the total score (r=0.375, p<0.05) (**Table 3**). **Table 4** shows the results of the hierarchical regression analysis. Model 1 shows a significant relationship between childhood trauma and the severity of depression. Model 2 shows a positive relationship between the addition of dissociative symptoms and an increased risk of depression (as evidenced by the increased explanatory power when DES scores were included in the model).

**Table 3:** Correlation between childhood trauma, dissociative experiences and depressive symptoms among the cases.

Scale Subscores	HAD Depression		Dissociative Experiences	
	r	p	r	p
Emotional Abuse	.342**	.03	.381**	.04
Physical Abuse	.241**	.02	.158**	.03
Sexual Abuse	.310**	.03	.256**	.04
Emotional Neglect	.252**	.02	.397**	.03
Physical Neglect	.281**	.04	.389**	.02
CTE Total	.442**	.03	.375**	.04

** p <.05.

**Table 4:** Determinants of HAD Depression score averages according to hierarchical regression analysis.

Model	B	SEM	β	t	p
1 Model	4.132	0.4687		8.245	<0.001
CTE	0.115	0.028	0.604	4.572	<0.001
2 Model	2.561	0.753		3.678	<0.001
CTE	0.030	0.048	0.174	0.745	0.409
DES	0.087	0.037	0.569	3.189	0.004

Model 1: F=72.44, SD=1.32, p<0.001. R2=0.458, Model 2: F=53.68, SD=2.37, p<0.001. R2=0.574, CTE: Childhood Trauma, DES: Dissociative experience scale, β: standardised regression coefficients, B: unstandardised regression coefficients.

## Discussion

The present study aimed to investigate the relationship between experiences of childhood abuse/neglect and the presence of depressive symptoms, dissociative experiences, and AR. The findings of this study revealed several important and clinically relevant observations. In terms of sociodemographic characteristics, we observed that AR was more common among women in our sample, and patients were often married and well-educated. We also found that the prevalence of AR tended to decline with age. In our study, nearly one-third (32.89%) of the AR patients were aged 20–30 years, consistent with previous reports that AR is more prevalent in younger adults (e.g., in their twenties and thirties) than in older populations (2, 21).

We found a statistically significant difference in mean scores of HADS-D and HADS-A scores between patients with AR and HCs. The AR patients, on average, exhibited higher levels of depression and anxiety symptoms than HCs. Moreover, our results provide evidence of a significant positive correlation between having AR and experiencing depressive symptoms (i.e., AR patients tended to have higher depression scores). Another important result of the present study is the statistically significant positive association between childhood trauma and depression scores in patients with AR. CTQ scores were moderately correlated with HADS-D scores, suggesting that greater exposure to childhood adverse experiences is associated with more severe depressive symptoms in these patients. Our finding, that higher cumulative trauma relates to higher depression, is supported by studies indicating that childhood traumatic experiences increase the risk of developing psychopathological symptoms and even future psychiatric disorders (22, 23).

In recent years, an increasing number of studies have focused on the role of childhood trauma in the etiology of various psychiatric disorders. Consistent associations have been demonstrated between early trauma and a range of mental health conditions, including somatic symptom disorders, sleep disorders, personality disorders, obsessive–compulsive disorder (OCD), anxiety disorders, and depression (24–26).

Our findings also showed that childhood trauma was significantly associated with dissociative experiences in AR patients. Specifically, we observed that higher CTQ levels (both total and in subcategories) were strongly associated with higher DES scores. This is consistent with the literature showing that childhood trauma experiences are linked to an increase in dissociative symptoms. Additionally, a meta-analytic review confirms a strong association between childhood abuse/neglect and later dissociation (27). Our study extends this knowledge by highlighting that even in a clinical population defined by a medical condition (AR), those with greater trauma histories tend to report more dissociative tendencies. On the other hand, previous research in non-clinical samples has found that obsessive–compulsive symptoms can be significantly associated with childhood traumatic experiences and dissociation levels (28). The concordance between these findings and the present results suggests that early-life traumatic experiences may represent a shared psychological vulnerability factor predisposing individuals to chronic otorhinolaryngological conditions, including allergic rhinitis, through long-term effects on emotional regulation and dissociative coping mechanisms.

Childhood trauma may also adversely affect the course and outcomes of serious mental illnesses. For example, one study reported that traumatic childhood experiences were associated with worse clinical outcomes in patients with major depressive disorder and bipolar disorder (29). In bipolar patients, a history of childhood trauma has been linked to more severe illness characteristics, such as earlier onset and greater symptom burden. There is also evidence that childhood maltreatment predicts a more unfavorable course of illness and treatment resistance in depression (30).

Understanding the mechanisms behind the trauma–depression link is important. Childhood abuse and neglect can disrupt neurobiological systems and psychosocial development, thereby increasing vulnerability to depression and other psychiatric disorders. For instance, early-life trauma can chronically activate the hypothalamic–pituitary–adrenal (HPA) axis and dysregulate stress-response systems (31).

Another important outcome of this study is the identification of a correlation between dissociative experiences in AR patients and childhood trauma and all its subcategories, namely physical neglect, emotional neglect, sexual abuse, emotional abuse, and physical abuse. Our data indicate that patients with AR who reported higher levels of childhood trauma tended to report more frequent dissociative symptoms. Our findings appear to be consistent with studies in the literature. Specifically, a meta-analysis showed that individuals who experienced abuse or neglect during childhood exhibited significant dissociative symptoms later in life (27).

Finally, our regression analysis revealed a relationship between childhood trauma, dissociation, and depression in AR patients. It appears that childhood trauma alone significantly contributes to the severity of depression, and the presence of dissociative symptoms further increases the risk of depression (as evidenced by the increased explanatory power when DES scores were added to the model). This suggests that dissociation may mediate or amplify the effect of trauma on depression. In practical terms, AR patients who have experienced childhood trauma and exhibit dissociative tendencies may be particularly vulnerable to developing depressive symptoms.

This study has several limitations. Firstly, the study was conducted at a single center, and the number of patients is insufficient for generalizing the findings to the general population. Therefore, multicenter studies with more participants are needed. Secondly, since this is a cross-sectional study, there are limitations in evaluating the findings longitudinally. Therefore, more longitudinal and case-control studies will more clearly evaluate the validity of our findings. Despite these limitations, our findings in this study will make a significant contribution to both the current literature and future studies in the field.

## Conclusion

To the best of our knowledge, this study is the first to provide evidence of a positive association between AR and childhood trauma and dissociative experiences. Furthermore, our results demonstrate that exposure to childhood trauma is associated with increased levels of depressive symptoms in patients with AR. These findings highlight the importance of considering a history of childhood trauma as a factor that may indirectly influence the clinical presentation of AR by triggering certain psychiatric disorders.

In clinical practice, the presence of significant childhood trauma in a patient diagnosed with AR necessitates a holistic approach. In these patients, psychiatric evaluation should not be neglected alongside medical treatment, and psycho-interventions addressing traumatic experiences (e.g., trauma-focused psychotherapy) should be prioritized. Furthermore, although a significant portion of AR patients in our sample exhibited depression, anxiety, and dissociative symptoms, they had not received any treatment. Therefore, actively identifying mental health issues in AR patients and employing psychotherapeutic approaches will positively impact AR treatment.

In summary, approaches combining psychiatric evaluation and psychotherapy with nasorhinopharyngeal treatment may be the most effective method for improving overall treatment outcomes in patients diagnosed with AR.

s) have reported that they did not receive any funding for the work presented in this article.

## Data Availability

The raw data supporting the conclusions of this article will be made available by the authors, without undue reservation.
